# Survival of patients with non-small cell lung cancer without treatment: a systematic review and meta-analysis

**DOI:** 10.1186/2046-4053-2-10

**Published:** 2013-02-04

**Authors:** Hesborn Wao, Rahul Mhaskar, Ambuj Kumar, Branko Miladinovic, Benjamin Djulbegovic

**Affiliations:** 1 Center for Evidence Based Medicine and Outcomes Research. Department of Internal Medicine, Morsani College of Medicine, University of South Florida Clinical and Translational Science Institute, 3515 East Fletcher Avenue, MDT 1202, Tampa, FL, 33612, USA; 2 Department of Internal Medicine, Division of Evidence-based Medicine and Health Outcomes Research University of South Florida, Tampa, FL, USA; 3 Departments of Hematology and Health Outcomes and Behavior, Moffitt Cancer Center & Research Institute, Tampa, FL, USA

**Keywords:** Best supportive care, Natural history, Meta-analysis, Palliative care, Placebo

## Abstract

**Background:**

Lung cancer is considered a terminal illness with a five-year survival rate of about 16%. Informed decision-making related to the management of a disease requires accurate prognosis of the disease with or without treatment. Despite the significance of disease prognosis in clinical decision-making, systematic assessment of prognosis in patients with lung cancer without treatment has not been performed. We conducted a systematic review and meta-analysis of the natural history of patients with confirmed diagnosis of lung cancer without active treatment, to provide evidence-based recommendations for practitioners on management decisions related to the disease. Specifically, we estimated overall survival when no anticancer therapy is provided.

**Methods:**

Relevant studies were identified by search of electronic databases and abstract proceedings, review of bibliographies of included articles, and contacting experts in the field. All prospective or retrospective studies assessing prognosis of lung cancer patients without treatment were eligible for inclusion. Data on mortality was extracted from all included studies. Pooled proportion of mortality was calculated as a back-transform of the weighted mean of the transformed proportions using the random-effects model. To perform meta-analysis of median survival, published methods were used to pool the estimates as mean and standard error under the random-effects model. Methodological quality of the studies was examined.

**Results:**

Seven cohort studies (4,418 patients) and 15 randomized controlled trials (1,031 patients) were included in the meta-analysis. All studies assessed mortality without treatment in patients with non-small cell lung cancer (NSCLC). The pooled proportion of mortality without treatment in cohort studies was 0.97 (95% CI: 0.96 to 0.99) and 0.96 in randomized controlled trials (95% CI: 0.94 to 0.98) over median study periods of eight and three years, respectively. When data from cohort and randomized controlled trials were combined, the pooled proportion of mortality was 0.97 (95% CI: 0.96 to 0.98). Test of interaction showed a statistically non-significant difference between subgroups of cohort and randomized controlled trials. The pooled mean survival for patients without anticancer treatment in cohort studies was 11.94 months (95% CI: 10.07 to 13.8) and 5.03 months (95% CI: 4.17 to 5.89) in RCTs. For the combined data (cohort studies and RCTs), the pooled mean survival was 7.15 months (95% CI: 5.87 to 8.42), with a statistically significant difference between the two designs. Overall, the studies were of moderate methodological quality.

**Conclusion:**

Systematic evaluation of evidence on prognosis of NSCLC without treatment shows that mortality is very high. Untreated lung cancer patients live on average for 7.15 months. Although limited by study design, these findings provide the basis for future trials to determine optimal expected improvement in mortality with innovative treatments.

## Background

Cancer is a major public health concern globally. It is the most frequent cause of death in economically developed countries [1]. Among all cancers, lung cancer is the leading cause of cancer deaths worldwide [2]. In the United States, approximately 221,130 new cases of lung cancer (14% of all cancer diagnoses) are expected in 2011 out of which 156,940 deaths (27% of cancer deaths) are estimated due to lung cancer [3]. Given the incurable nature of lung cancer, it is considered a terminal illness with a five-year survival rate of approximately 16% [3].

Patients diagnosed with terminal illness such as lung cancer confront several decisions related to management of the disease. Opting for treatment (for example, chemotherapy, radiotherapy, or surgery) instead of palliation, or *vice versa,* is one such critical decision. Depending on the stage of the disease, potential benefits of anticancer therapy intended to palliate specific tumor-related symptoms may be at the expense of treatment-related harms and the inconvenience associated with undergoing treatment. At other times, palliative care (for example, pain medications or low dose radiotherapy) [4] rather than anticancer therapy may be preferable. Informed decision-making related to the management of a terminal disease thus requires accurate prognosis of the disease with or without treatment.

Briefly, prognosis refers to the likelihood of an individual developing a particular health outcome over a given period of time, based on the individual’s clinical and non-clinical profile [5]. Accurate assessment of prognosis is key to informed decision-making. For example, if a patient is diagnosed with a terminal illness such as lung cancer, a prognostic question of critical concern to the patient, family, and the physician is how long the patient is expected to live. Other important outcomes may include disease progression, health-related quality of life, and treatment-related harms. Reliable prognostication of life expectancy can prevent subjecting patients to costly and unnecessary treatment for an unduly long period before transitioning to hospice care [6]. This in turn can help patients and their families prepare for the impending events and plan for the patient’s remaining lifespan [7]. Accurate prognostic information can also help physicians decide on choice of curative versus palliative treatments. For instance, if evidence shows no effect of curative treatment on disease progression, significant treatment-related harms can be avoided in favor of palliative treatments [7]. It can help investigators avoid optimism bias, the ‘unwarranted belief in the efficacy of new therapies’ [8] or making ‘overly optimistic assumptions regarding treatment benefits when designing RCTs’ [9]. Accurate disease prognosis thus underpins all management decisions related to the disease, including choice of treatment, planning of supportive care, as well as allocation of resources.

Despite the significance of disease prognosis in clinical decision-making, systematic assessment of prognosis in patients with lung cancer without treatment has not been performed. We are aware of only one narrative review on the subject [4,10]. Accordingly, this systematic review was undertaken to assess the survival of patients with a confirmed diagnosis of lung cancer without active treatment. Specifically, our aim was to estimate overall survival in lung cancer when no anticancer therapy is provided.

## Methods

This systematic review was conducted as per the methods elaborated in a protocol that was developed *a priori.* The results are reported according to PRISMA (Preferred Reporting Items for Systematic Reviews and Meta-Analyses) statement [11]. An ideal study design to assess the natural history of a terminal disease such as lung cancer is a cohort study. Specifically, an inception cohort, whereby a well-defined group of patients at the same disease stage is assembled at first diagnosis and followed for a defined period of time [12-14]. However, given the availability of treatments for lung cancer in recent years, it would be unethical and logistically challenging to conduct such a study. An alternative approach is to assess prognosis from retrospective lung cancer registries, case series or from the control arm of individual RCTs that compare active treatment with either no treatment, placebo, or best supportive care [5,15].

### Study eligibility

In this review, any retrospective or prospective cohort study assessing prognosis in lung cancer without treatment and any RCT assessing the role of treatment versus no treatment, were eligible for inclusion. A study was eligible for inclusion irrespective of language or publication type.

### Search strategy

We conducted a systematic search of MEDLINE and Cochrane library electronic databases, proceedings of major scientific meetings, and bibliographies of eligible studies to identify all relevant studies. To retrieve lung cancer prognosis studies in PubMed, we employed search strategies suggested by Wilczynski [16] that optimizes search sensitivity and specificity. Search details used included: ("lung neoplasms" [MeSH Terms] AND "prognosis" [All Fields] AND "cohort" [All Fields] AND "mortality" [Subheading] OR "natural course" [All Fields] OR "mortality" [All Fields] OR "survival" [All Fields] OR "survival" [MeSH Terms]). To retrieve RCTs in PubMed, we employed strategies suggested by Haynes [17] with the following search details: ("lung neoplasms" [MeSH Terms] AND "randomized controlled trial" [Publication Type]) AND ("palliative care" [All Fields] OR "hospice care" [All Fields] OR "supportive care" [All Fields] OR "best supportive care" [All Fields] OR "placebo" [All Fields] OR "symptomatic treatment" [All Fields] OR "no chemotherapy" [All Fields] OR "no treatment" [All Fields]). In the Cochrane library, we utilized a free text search using the term “Lung cancer” to identify RCTs focusing on lung cancer. We manually searched abstracts of the American Society of Clinical Oncology and American Society of Hematology meetings and utilized the snowballing procedure to identify other relevant studies. Studies published until June 2011 were included. No restrictions were made regarding the language of the publication.

### Inclusion and exclusion criteria

A prospective or retrospective cohort study assessing overall survival as an outcome in lung cancer patients without treatment was eligible for inclusion. A RCT was included if it enrolled patients with confirmed diagnosis of lung cancer, compared treatment versus no treatment (for example, supportive care, best supportive care, palliative care, placebo, and so on), and assessed overall survival as an outcome. A study in which patients had anticancer treatment prior to enrollment and subgroup analyses were excluded. Additionally, RCTs comparing two active treatments were excluded. Two reviewers read the titles and abstracts of identified citations to identify potentially eligible studies. Full text of potentially relevant reports were retrieved and examined for eligibility. Disagreements about study inclusion or exclusion were resolved via discussion until a consensus was reached.

### Data extraction

Data extraction was performed using a standardized data extraction form. Two reviewers independently extracted the following information from each included study: number of patients enrolled, number of deaths, median survival, funding source (industry versus public, and so on), type of centers involved (single versus multicenter, and so on.), patient demographics, patients’ baseline clinical characteristics, and type of control arm (for RCTs only). For cohort studies, we extracted data on the number of deaths and total number of patients diagnosed with lung cancer. For RCTs, we extracted data on the number of deaths (all-cause mortality) and number of participants randomized to the control arm.

### Assessment of methodological quality

To evaluate the methodological quality of included studies, a modified checklist of predefined criteria was developed on four methodological domains pertinent to minimization of bias. This modified checklist uses applicable elements from existing tools (Quality in Prognosis Studies tool [18], Evidence-Based Medicine Group criteria for prognostic studies [19], Newcastle-Ottawa Quality Assessment Scale [20], Cochrane Collaboration risk of bias criteria [21]) and related studies (Hudak *et al*. [22] and Altman [23]). The four domains included ‘participation bias’ (extent to which study sample represents the population of interest on key characteristics), ‘attrition bias’ (extent to which loss to follow-up of the sample was not associated with key characteristics), ‘outcome measurement’ (extent to which outcome of interest is adequately measured in study participants), ‘data analysis’ and ‘reporting’ (extent to which statistical analysis and data reporting are appropriate for the study design). The modified checklist contains 11 items for cohort studies and 14 items for RCTs. For each item, a study either fulfilled a certain criterion (scored ‘Yes’) or failed to fulfill the criterion (scored ‘No’). To assess methodological quality of the studies included, we focused on the proportion that fulfilled each quality criterion (Table 1).

**Table 1:** Methodological quality of lung cancer prognosis studies

**Study Design/Domain/Criterion**		**Criteria fulfilled**	
		**n/N**	**%**
**Cohort studies** (11 items)			
	**Participation bias**		
A	Population of interest is adequately described for key characteristics [15]	7/7	100
B	Study setting and geographic location is adequately described [15]	7/7	100
C	Baseline sample is adequately described for key characteristics [15]	4/7	57
D	Inclusion and exclusion criteria are adequately described [15]	5/7	71
E	There is adequate participation in the study by all eligible patients [15]	7/7	100
	**Attrition bias**		
F	Follow-up is sufficiently long for outcome to occur (≥6 months) [16,18,19,46]	6/7	86
G	Patients with missing data were reported [15,17]	7/7	100
	**Outcome measurement**		
H	Definition of outcome is provided *a priori*[15]	7/7	100
I	Objective definition of outcome is provided [15,16,18,19]	7/7	100
	**Data analysis and reporting**		
J	Alpha error and/or beta error is specified *a priori*	2/7	29
K	Frequencies of most important data (for example, outcomes) are presented [18,19,47]	7/7	100
**Randomized controlled trials** (14 items)			
	**Participation bias**		
L	Population of interest is adequately described for key characteristics [15]	15/15	100
M	Study setting and geographic location is adequately described [15]	7/15	47
N	Baseline sample is adequately described for key characteristics [15]	14/15	93
O	Inclusion and exclusion criteria are adequately described [15]	14/15	93
P	Patients were balanced in all aspects except the intervention	15/15	93
	**Attrition bias**		
Q	Follow-up is sufficiently long for outcome to occur (≥6 months) [16,18,19,46,48]	8/15	53
R	Proportion of sample completing the study is adequate (≥80%) [15,16,18,47,49,50]	9/15	60
S	Description of withdrawal (incomplete outcome data) is provided [15,17]	15/15	100
T	Characteristics of drop-outs versus completers is provided [15]	2/15	13
	**Outcome measurement**		
U	Definition of outcome is provided *a priori*[15]	15/15	100
V	Objective definition of outcome is provided [15,16,18,19]	15/15	100
	**Data analysis and reporting**		
W	Alpha error and/or beta error is specified *a priori*	7/15	47
X	Data analysis was based on intention-to-treat analysis principle [17]	9/15	53
Y	Frequencies of most important data (for example, outcomes) are presented [18,19,47]	15/15	100

### Statistical analysis

Data synthesis was conducted according to the study design separately as well as combined in the final stage (that is, retrospective cohort and RCT). For the purpose of meta-analysis, we used methods by Stuarts *et al*. [24] to transform the proportions into a quantity according to the Freeman-Tukey variant of the arcsine square root transformed proportion. The pooled proportion was calculated as a back-transform of the weighted mean of the transformed proportions, using the random-effects model. To perform meta-analysis of median survival, we used published methods [25] to pool the estimates as mean survival and standard error under the random effects model. That is, using median survival and range reported in Kaplan-Meier curve, we converted these estimates into mean survival and standard error. Heterogeneity of treatment effects between trials was assessed using the I-squared statistic [21] with the following thresholds for I-squared statistic values: low (25% to 49%), moderate (50% to 74%), and high (≥75%) [26]. We explored the potential causes of heterogeneity by assessing the differences between subgroups using the test of interaction. We assessed robustness of the results by conducting sensitivity analysis with respect to methodological quality criteria of reporting, study location, and funding source. RevMan Version 5.1 [27] was used to perform the analyses.

## Results

### Literature search

**Figure 1:**
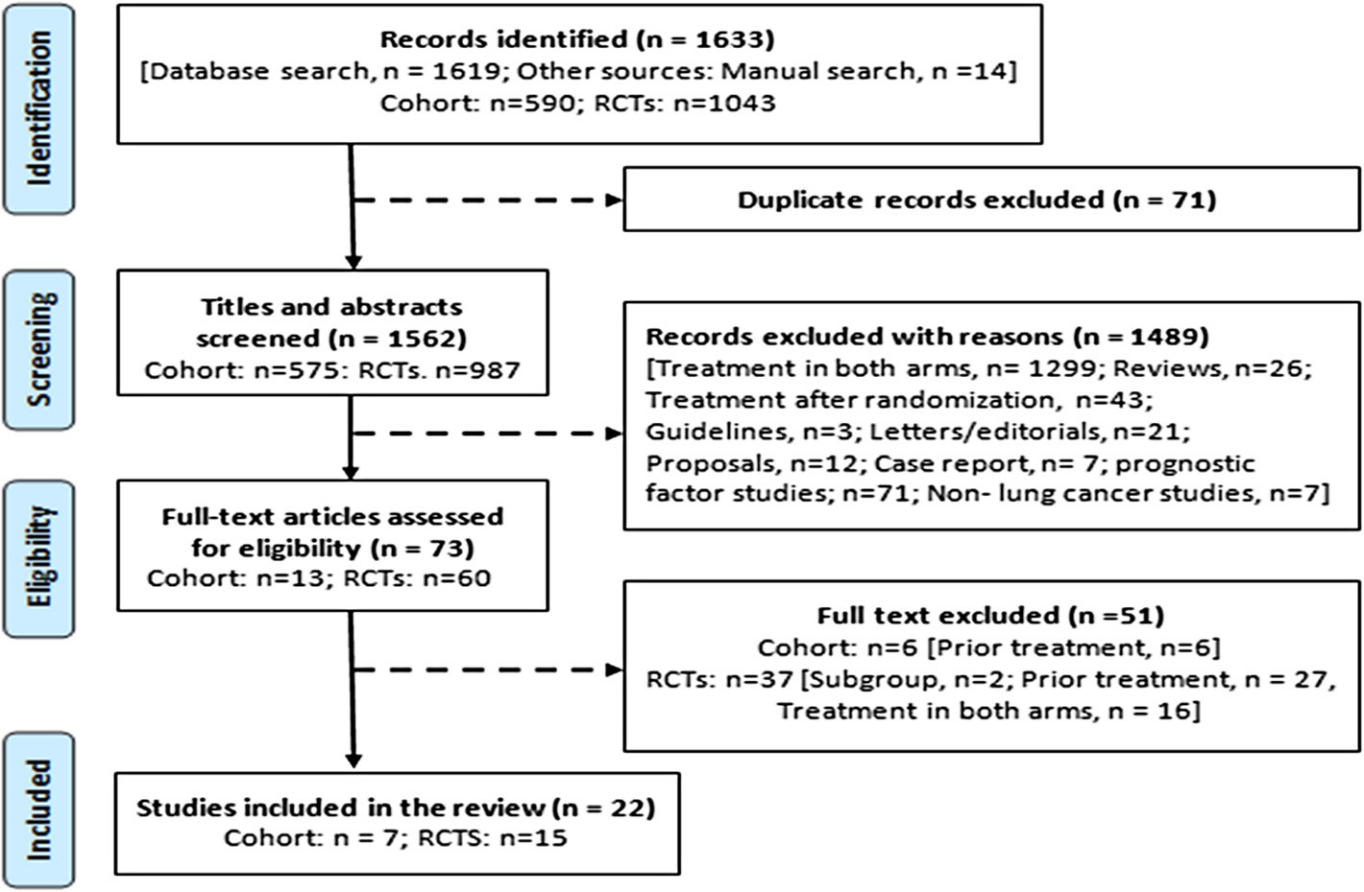
A flow diagram depicting the literature search process.

A flow diagram depicting the literature search process based on PRISMA [11] is shown in Figure 1. Initial search identified 1,562 potentially relevant citations excluding 71 duplicates. After initial screening of titles and abstracts, 1,489 records were not relevant for reasons depicted in Figure 1 and were excluded. Further assessment of full texts of remaining 73 studies led to exclusion of 51 studies. Altogether, 22 studies met the pre-defined inclusion criteria: 7 were retrospective cohort studies [20,28-33] and 15 were RCTs [34-48].

### Study characteristics

We did not find any inception cohort study or prospective cohort study assessing prognosis of patients with lung cancer without treatment. The seven retrospective cohort studies included 4,418 patients and the fifteen RCTs enrolled 1,031 patients. Altogether, the 22 studies included 5,449 patients. All studies assessed prognosis in patients with NSCLC and were published between 1973 and 2009 (Table 2).

**Table 2:** Characteristics of studies included in the review

**Study**	**N**	**Study period (years)**	**Disease Stage**		**Histology**			**Male**	**Median Age (years)**
			**I**	**II**	**squamous**	**adeno**	**large-cell**		
**(a) Cohort studies**									
Raz 2007	1432	13	1432	NR	460	419	89	747	74
Wisnivesky 2007^a^	2344	8	NR	NR	NR	NR	NR	1292	NR
Chadha 2005	39	11	23	13	18	88	5	4	77
Henschke 2003	131	7	131	NR	NR	NR	NR	NR	NR
McGarry 2002^a^	49	5	NR	NR	NR	NR	NR	49	NR
Vrdoljak 1994	130	7	55	56	61	35	34	120	60
Hyde 1973	293	8	NR	NR	NR	NR	NR	NR	NR
Total/(Range)	4418	(5 to 13)	1641	68	539	542	128	2211	
**(b) RCTs**			**III**	**IV**					
Goss 2009^c^	101	2 (0.23)	17	84	25	46	11	61	76
Anderson 2000	150	2	92	58	NR	NR	NR	91	64
ELVIS 1999^c^	78	1 (1.08)	22	56	33	29	3	69	74^b^
Cullen 1999^c^	176	8 (2.17)	88	88	103	42	6	122	64
Thongprasert 1999	98	4	49	49	31	49	12	NR	60
Helsing 1998^c^	26	5 (3.33)	3	23	5	17	4	18	65
Cartei 1993	50	7	NR	50	25	17	8	36	57
Leung 1992^c^	66	4 (3.58)	58	NR	31	18	7	48	62
Cellerino 1991	61	3	61	NR	38	18	5	59	62
Quoix 1991	22	3	NR	22	NR	NR	NR	NR	NR
Kaasa 1991	43	3	NR	43	16	16	11	31	62^b^
Ganz 1989	26	2	NR	26	9	17	NR	23	NR
Rapp 1988	50	3	50	NR	12	24	12	38	58
Cormier 1982	17	2	17	NR	8	2	6	16	60
Laing 1975	67	2	15	20	23	5	9	59	64
Total/(Range)	1031	(1 to 8)	472	519	359	300	94	671	(57 to 76)

N = Sample size or number of participants enrolled; NR = data not reported; adeno**,** = adenocarcinoma; squamous, = squamous cell carcinoma; large-cell, = large-cell carcinoma; ^a^sample includes stage I and II cancer; ^b^recorded mean age where median age was not reported or not extractable^,^^c^median follow-up in parenthesis.

#### Cohort studies

The median sample size in the cohort studies was 131 patients (range: 39 to 2,344 patients) with a median study period of 8 years (range: 5 to 13 years). Fifty-seven percent (4/7) and 29% (2/7) of the studies reported number of patients with stage I and stage II NSCLC, respectively. Forty-three percent (3/7) of the studies reported patients’ cancer histology. Seventy-one percent (6/7) of the studies reported patient’s gender. Forty-three percent (3/7) of the studies reported median age. Forty-three percent (3/7) of the studies were conducted at single institutions, 43% (3/7) were at multicenter national institutions, and 14% (1/7) of the studies had unspecified study location. Twenty-nine percent (2/7) of the studies were publicly funded, 14% (1/7) were funded by both public and industry, and 57% (4/7) had not specified funding sources.

#### RCTs

The median number of patients enrolled in the RCTs was 61 patients (range: 17 to 176 patients) with a median study period of 3 years (range: 1 to 7 years). Median follow-up was reported in 33% (5/15 of RCTs) and ranged between 2.7 and 43 months. Seventy-three percent (11/15) of the studies reported number of patients with stage III/IV NSCLC. Seventy-three percent (13/15) of the studies reported patients’ cancer histology. Eighty-seven percent (13/15) of the RCTs reported patient’s gender and median age. Twenty percent (3/15) of the RCTs were conducted at single institutions, 27% (4/15) were multicenter national studies, 20% (3/15) were multicenter international studies, and 33% (5/15) did not specify study locations. Seven percent (1/15) of the RCTs were funded by the public, 33% (5/15) by industry, 7% (1/15) by both the public and industry, and 53% (8/15) had unspecified funding sources.

#### Types of control in RCTs

Three studies described *best supportive care* as comprising ‘symptomatic or palliative treatment excluding chemotherapy’ [49], ‘palliative radiotherapy, antibiotics, and corticosteroids’ [35], ‘palliative radiotherapy, opioid analgesics, and psychosocial support’ [42], or ‘radiation therapy, pain medication, nutritional and psychological support, thoracocentesis and/or tube thorascopy’ [48]. Three studies described *supportive care* as comprising ‘analgesics, an antitussive, relief of increased intracranial pressure, palliative radiotherapy, treatment of infections and pleural effusions’ [35], ‘symptomatic irradiation to involved fields’ [36], or ‘palliative radiation, analgesics, and psychosocial/nutritional support’ [40]. *Palliative care* consisted of ‘radiotherapy, antibiotics, cough suppressants, and analgesics’ [38]. *Symptomatic treatment* included ‘glucocorticosteroids and anabolic steroids’ [43]. No descriptions were provided for *placebo* and ‘*no treatment*’.

### Methodological quality

#### Cohort

All seven cohort studies fulfilled 64% (7/11) of the quality criteria (Table 1). That is, adequate description of the population of interest for key characteristics, adequate description of study setting/geographic location, adequate participation in the study by all eligible patients, reporting of patients with missing data, *a priori* and objective definition of outcomes, and presentation of frequencies of most important data (for example, outcome) were reported in all studies. However, baseline sample was adequately described for key characteristics in 57% (4/7) of the studies, inclusion and exclusion criteria were adequately described in 71% (5/7) of the studies, follow-up was sufficiently long for outcome to occur in 86% (6/7) of the studies, and alpha error and/or beta error were specified *a priori* in 29% (2/7) of the studies.

#### RCTs

All 15 RCTs fulfilled 36% (5/14) of the quality criteria (Table 1). That is, adequate description of the population of interest for key characteristics, adequate description of withdrawal (incomplete outcome data), *a priori* and objective definition of outcomes, and frequencies of most important data were reported in all RCTs. However, study setting and geographic location were adequately described in 47% (7/15) of the RCTs, baseline sample was adequately described for key characteristics in 93% (14/15) of the RCTs, inclusion and exclusion criteria were adequately described in 93% (14/15) of the RCTs, patients were balanced in all aspects except the intervention in 93% (14/15) of the RCTs, follow-up was sufficiently long for outcome to occur in 53% (8/15) of the RCTs, proportion of sample completing the study was adequate in 60% (9/15) of the RCTs, characteristics of drop-outs versus completers was provided in 13% (2/15) of the RCTs, alpha error and/or beta error was specified *a priori* in 47% (7/15) of the RCTs, and data analysis was based on intention-to-treat analysis principle in 53% (9/15) of the RCTs.

### Mortality

#### Cohort

**Figure 2:**
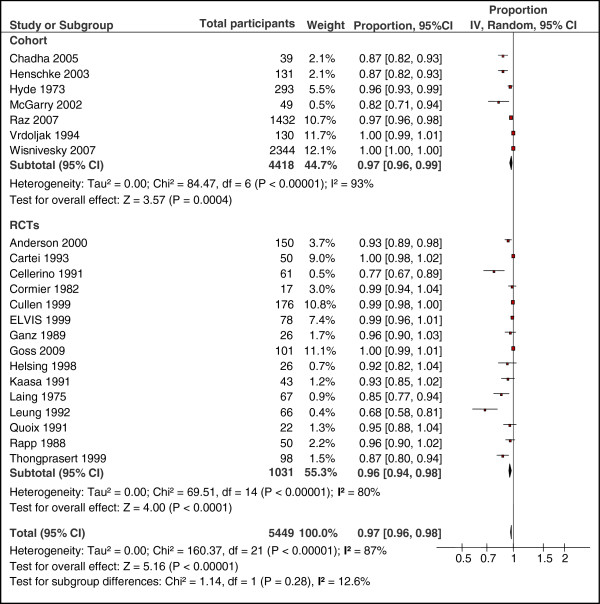
**Pooled proportion of mortality in lung cancer studies.** The size of each square is proportional to the weight of the study (inverse variance).

Data on mortality was extractable from all seven cohort studies enrolling 4,418 patients. As shown in Figure 2, the pooled proportion of mortality for patients without anticancer treatment was 0.97 (95% CI: 0.96 to 0.99). There was a statistically significant heterogeneity among pooled cohort studies (I-squared = 93%, *P* <0.00001).

#### RCTs

Data on mortality was extractable from the control arm of all 15 RCTs (1,031 patients). Figure 2 shows that the pooled proportion of mortality for patients in the control arm (without active treatment) was 0.96 (95% CI: 0.94 to 0.98). There was a statistically significant heterogeneity among pooled control arm of RCTs (I-squared = 80%, *P* <0.00001).

#### Combined (Cohort and RCTs)

Pooled proportion of mortality across the 22 studies was 0.97 (95% CI: 0.96 to 0.98). Because these two designs are inherently different from each other, we conducted separate analyses. However, as shown in Figure 2, testing for subgroup differences showed no statistically significant heterogeneity between the two study designs (*P* = 0.28).

### Median survival

#### Cohort

**Figure 3:**
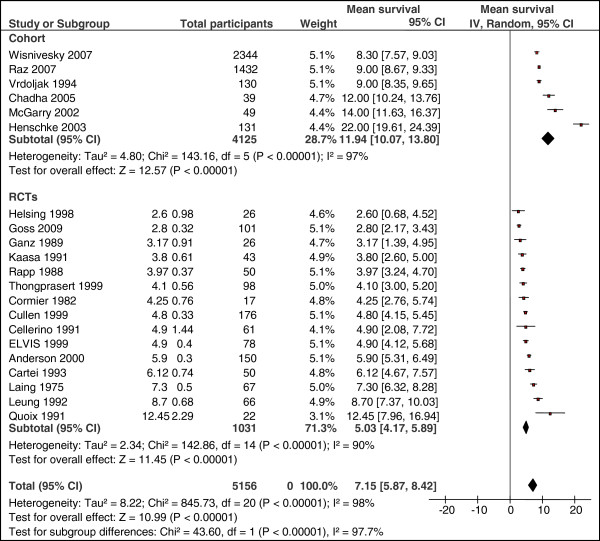
**Pooled mean survival and heterogeneity between subgroups.** The size of each square is proportional to the weight of the study (inverse variance).

Data on median overall survival was extractable from six cohort studies (4,125 patients). As shown in Figure 3, the pooled mean survival was 11.94 months (95% CI: 10.07 to 13.8). There was a statistically significant heterogeneity among pooled cohort studies (I-squared = 97%, *P* <0.00001).

#### RCTs

Data on median overall survival was extractable from all 15 RCTs (1,031 patients). The pooled mean survival for patients in the control arm was 5.03 months (95% CI: 4.17 to 5.89) (Figure 3). There was a statistically significant heterogeneity among pooled control arm of RCTs (I-squared = 90%, *P* <0.00001).

#### Combined (Cohort and RCTs)

Pooled proportion of mean survival across the 21 studies was 7.15 months (95% CI: 5.87 to 8.42). Test for subgroup differences showed statistically significant heterogeneity between the two study designs (I-squared = 97.7%, *P* <0.00001). Thus, the mean survival was influenced by study design (Figure 3).

### Sensitivity analysis

**Figure 4:**
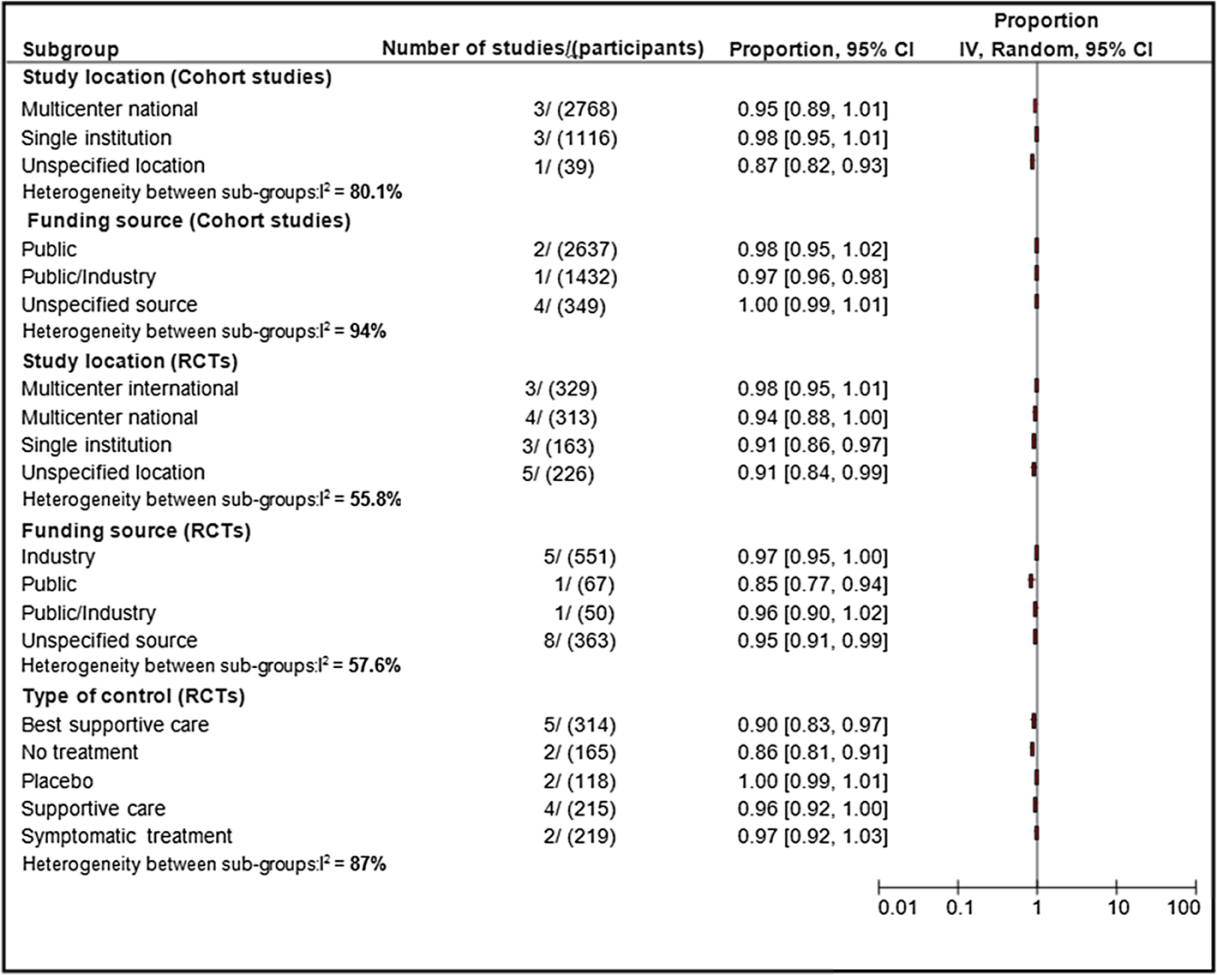
**Pooled proportions of mortality and heterogeneity between subgroups.** The size of each square is proportional to the weight of the study (inverse variance).

To assess the robustness of overall results according to the study design (cohort versus RCT) as well as explore the reasons for observed heterogeneity in the pooled proportion of mortality and mean survival, we conducted additional sensitivity analyses. For both cohort studies and RCTs, we conducted sensitivity analyses according to methodological quality criteria, funding source, and study location. For RCTs only, we conducted additional sensitivity analyses according to type of control. The results of sensitivity analyses are summarized in Figure 4. Overall, the results remained unchanged in the sensitivity analyses. There were no statistically significant differences in the proportion of mortality.

#### Cohort

In cohort studies, there was no statistically significant difference in the proportion of mortality according to any methodological criteria of reporting. With respect to study location, the pooled proportion of mortality in cohort studies conducted at multicenter national locations was 0.95 (95% CI: 0.89 to 1.01) and at single institution was 0.98 (95% CI: 0.95 to 1.01) whereas the pooled proportion of mortality in cohort studies conducted at unspecified locations was 0.87 (95% CI: 0.82 to 0.93). Test for overall interaction among these subgroups was statistically significant (*P* = 0.007). Regarding funding source, the pooled proportion of mortality in public-funded, unspecified funding sources, and public/industry-funded cohort studies were 1.00 (95% CI: 1.00 to 1.00), 1.00 (95% CI: 0.99 to 1.00), and 0.97 (95% CI: 0.96 to 0.98), respectively. The test for overall interaction among these subgroups was statistically significant (*P* <0.0001).

#### RCTs

There was no statistically significant difference in the proportion of mortality according to methodological criteria of reporting, study location, and funding source. With respect to type of control, the pooled proportion of mortality in RCTs involving best supportive care, no treatment, placebo, supportive care, and symptomatic treatment as control were 0.90 (95% CI: 0.83 to 0.97) and in RCTs involving supportive care as control was 0.96 (95% CI: 0.92 to 1.00), 0.86 (95% CI: 0.81 to 0.92), 1.00 (95% CI: 0.99 to 1.01), 0.96 (95% CI: 0.92 to 1.00), and 0.97 (95% CI: 0.92 to 1.03), respectively. Test for overall interaction among these subgroups was statistically significant (*P* <0.00001).

We considered performing subgroup analysis based on median follow-up. However, only one cohort study [32] and five RCTs [38,39,41,42,45] reported these data. The median follow-up in the cohort study was 40 months whereas in the RCTs, the median follow-up was 2.7, 13, 26, 40, and 40 months, respectively. Given that survival of patients with cancer differs by stage, we considered performing analysis by cancer stage (I, II, III, versus IV). However, only two cohort studies (29%) and two RCTs (13%) reported data by stage. Thus, it was not possible to perform meta-analysis based on the four stages.

## Discussion

This is the first study to provide the most comprehensive data related to survival of lung cancer patients. The results show that prognosis of patients with lung cancer not receiving treatment is very high. Regardless of the study design (that is, cohort versus RCTs) the findings were similar and did not differ according to disease severity. For example, all cohort studies assessed mortality in patients with early stage NSCLC (stage I/II) and all RCTs enrolled patients with advance stage NSCLC (stage III/IV). However, the mortality rates from cohort and RCTs essentially remained unchanged (97% versus 96%). Overall, included studies were of moderate methodological quality.

The findings from our study are similar to the study by Detterbeck and Gibson [4] which showed a 98% five-year mortality rate for stage I/II lung cancer (median survival = 10 months). Despite the obvious similarity in results our study is significantly different in the conduct and analysis. For example, the study by Detterbeck and Gibson [4] did not employ a systematic approach to data collection and analysis (that is, not a systematic review) and therefore the findings are not reproducible. The similarity in findings might be an artifact of play of chance. Furthermore, quantitative synthesis of results across included studies was not performed in the study by Detterbeck and Gibson [4] which was undertaken in our study. Another unique feature of our study lies in the inclusion of RCTs in addition to retrospective studies. None of the previous studies on the topic have utilized the approach of pooling data from one arm of RCTs for accurate assessment of prognosis. Therefore, due to the reasons enumerated, the study presented here is the most comprehensive to date in reporting survival of NSCLC patients without treatment.

Our study has some limitations. For example, we observed a statistically significant heterogeneity in pooled results which we could not explain through subgroup analyses. We suspect that the observed heterogeneity is clinical and not methodological. Specifically in the case of RCTs, the constitution of control arm varied across pooled studies. For example, five RCTs employed best supportive care as control, four had supportive care, two had placebo, two had no treatment and another two had symptomatic treatment as control. While, the definitions are very clear on placebo and no treatment, which was also explained by the sensitivity analyses (I-squared = 0% for both subgroups), the composition of best supportive care, supportive care, and symptomatic treatment varied significantly across pooled studies. In these cases, the observed heterogeneity remained unexplained. Also, whereas a significant number of studies (11 of 15 RCTs) included had some form of treatment even if used for the purpose of symptom palliation, we were unable to assess the effect of the supportive treatment on survival based on available data. Thus, the clinical heterogeneity may be attributed to stage of disease and/or differential therapies. The studies included had different follow-up periods, however, due to limited data reported, we were unable to perform subgroup analysis based on median follow-up. How much this difference accounts for results is thus not known. It is also unclear whether results would have changed had we performed the analysis by cancer stages (I, II, III, versus IV) as opposed to by stage I/II and III/IV. The former was not possible due to the limited data reported. Because studies included enrolled patients with NSCLC, our results may not entirely apply to all lung cancer patients. However, it is important to note that a systematic review is limited by the availability of data and we did include all available data related to prognosis of NSCLC patients without treatment.

## Conclusion

The aim of this review was to estimate overall survival (natural history) in lung cancer when no anticancer therapy is provided. Our study shows that untreated lung cancer patients live on average for 7.15 months (95% CI: 5.87 to 8.42). Comprehensive data on the natural history of lung cancer is required for informed decision making by patients, physicians and researchers. For patients, it serves as the basis for their expected outcome with and without treatment, which is critical in cases of diseases with high mortality. For physicians, accurate and reliable information facilitates shared decision making with patients, related to choice of interventions or no intervention. Most importantly, the findings are needed by researchers to avoid optimism bias [8]. A study by Djulbegovic *et al*. [8] assessed the role of optimism bias in a cohort of trials conducted by the National Cancer Institute Cooperative Groups and concluded that optimism bias is the primary reason for inconclusive findings in the context of RCTs. Similarly, a systematic review by Gan and colleagues [9] showed that investigators tend to make overly optimistic assumptions regarding treatment benefits when designing RCTs. Accordingly, the results from our study will help researchers determine the most optimal rate of expected improvement in mortality with innovative/newer treatments.

## Abbreviations

CI: Confidence interval; NSCLC: Non-small cell lung cancer; PRISMA: Preferred Reporting Items for Systematic Reviews and Meta-Analyses; RCT: Randomized controlled trial.

## Competing interests

The authors declare that they have no competing interests.

## Authors’ contributions

HW and RM were responsible for the study’s conception and design. HW acquired, analyzed and interpreted the data. HW drafted the manuscript while all other authors revised it critically for important intellectual content before giving approval of the final version to be published. All authors read and approved the final manuscript.
